# Surgery for spontaneous intracerebral hemorrhage

**DOI:** 10.1186/s13054-020-2749-2

**Published:** 2020-02-07

**Authors:** Airton Leonardo de Oliveira Manoel

**Affiliations:** 1 Department of Critical Care Medicine, Hospital Alemão Oswaldo Cruz, São Paulo, Brazil; 2 Department of Critical Care Medicine, Neurocritical Care Unit, Hospital Santa Paula, São Paulo, Brazil

**Keywords:** Stroke, Intracerebral hemorrhage, Hypertensive intracerebral hemorrhage, Neurosurgical procedures, STICH, MISTIE, Glasgow outcome scale

## Abstract

Spontaneous intracerebral hemorrhage is a devastating disease, accounting for 10 to 15% of all types of stroke; however, it is associated with disproportionally higher rates of mortality and disability. Despite significant progress in the acute management of these patients, the ideal surgical management is still to be determined. Surgical hematoma drainage has many theoretical benefits, such as the prevention of mass effect and cerebral herniation, reduction in intracranial pressure, and the decrease of excitotoxicity and neurotoxicity of blood products.

Several surgical techniques have been considered, such as open craniotomy, decompressive craniectomy, neuroendoscopy, and minimally invasive catheter evacuation followed by thrombolysis. Open craniotomy is the most studied approach in this clinical scenario, the first randomized controlled trial dating from the early 1960s. Since then, a large number of studies have been published, which included two large, well-designed, well-powered, multicenter, multinational, randomized clinical trials. These studies, The International Surgical Trial in Intracerebral Hemorrhage (STICH), and the STICH II have shown no clinical benefit for early surgical evacuation of intraparenchymal hematoma in patients with spontaneous supratentorial hemorrhage when compared with best medical management plus delayed surgery if necessary. However, the results of STICH trials may not be generalizable, because of the high rates of patients’ crossover from medical management to the surgical group. Without these high crossover percentages, the rates of unfavorable outcome and death with conservative management would have been higher. Additionally, comatose patients and patients at risk of cerebral herniation were not included. In these cases, surgery may be lifesaving, which prevented those patients of being enrolled in such trials. This article reviews the clinical evidence of surgical hematoma evacuation, and its role to decrease mortality and improve long-term functional outcome after spontaneous intracerebral hemorrhage.

## Introduction

Spontaneous intracranial hemorrhage (ICH), i.e., nontraumatic hemorrhage into the brain parenchyma ± ventricles, is a severe type of stroke with high mortality rates [1]. Systemic arterial hypertension and cerebral amyloid angiopathy represent the two main risk factors of primary ICH [2, 3].

While our understanding of this severe neurological disease has developed in the last years, there is no specific treatment that has been shown to improve outcome. Therapies targeting hematoma expansion, such as the aggressive reduction in blood pressure [target systolic blood pressure (SBP) < 140 mmHg] [4, 5], the administration of tranexamic acid, and the use of recombinant activated factor VII [6, 7] have failed to improve functional outcome.

Intuitively, hematoma evacuation may have therapeutic potential, mainly based on the theoretical advantages of preventing or correcting the acute effects of hematoma and its blood products into the surrounding healthy brain parenchyma. However, because the most common sites of spontaneous ICH are the deep brain structures, such as the basal ganglia and the thalamus, a large layer of brain tissue must be crossed during surgery, which may cause iatrogenic damage of healthy cerebral tissue. Additionally, neurosurgical procedures are not free of risks and adverse effects. Postsurgical complications (e.g., hemorrhages and infections) are not uncommon in this clinical scenario and carry high rates of morbidity and mortality [8].

Several surgical and invasive approaches exist concerning monitoring strategies, clot removal and mass effect control. Open craniotomy is the most studied approach in this clinical scenario, but other surgical approaches, such decompressive craniectomy ± hematoma drainage, image-guided stereotactic endoscopic aspiration, and minimally invasive catheter evacuation followed by thrombolysis, have also been attempted. None of these surgical techniques have improved clinical outcome when compared to best medical management.

However, large lobar hemorrhages or hematomas in the posterior fossa may lead to life-threatening cerebral or brainstem herniation, which may require life-saving emergent surgical evacuation. In such clinical scenarios, best medical management is probably not equipoised with surgery, which prevents the inclusion of these patients in a randomized clinical trial. This review summarizes the current literature on the surgical management of ICH, and its possible role to decrease mortality and improve long-term functional outcome.

### Search strategy

A PubMed search for articles published from inception to July 2019 was performed by using the terms “Spontaneous Intracerebral Hemorrhage” [Mesh] AND “Surgery” [Mesh], which returned 261 articles. Also, the reference lists of the most recent guidelines on the management of ICH were scrutinized [9]. The author’s database was also searched for additional articles.

### Mechanisms of brain injury and the hyperacute management after intracerebral hemorrhage

The mechanisms responsible for brain injury within the cerebral hematoma and the surrounding tissues are multiple and complex, which includes the primary effects of blood into the brain parenchyma and the secondary effects of hemoglobin breakdown and its products. Initially, there is the direct effect of acute hemorrhage into the brain parenchyma, causing disruption and mass effect within the cerebral tissue. This primary brain injury is followed by the interruption of bleeding in approximately two thirds of patients. However, in the remaining one third of patients, hematoma continues to expand in the first 24 h, which contributes to additional mass effect, midline shift [10], leading to further neurological deterioration and an increased risk of unfavorable outcome [11, 12].

The hyperacute management of ICH is focus on patients’ airway, breathing, and circulation stabilization, followed by the prevention of hematoma expansion. Several therapies attempting to reduce hematoma expansion have been studied, such as early aggressive blood pressure control [4, 5], the administration of tranexamic acid [6], and the use of recombinant activated factor VII [6, 7]. The use of recombinant activated factor VII reduced hematoma growth but did not decrease mortality or improve functional outcome [7]. Likewise, the early use of tranexamic acid was associated with a significant reduction in hematoma expansion, but did not improve functional outcome at 90 days [6]. Blood pressure control in the acute phase has modest effect in reducing hematoma growth; however, a preplanned pooled analysis of individual patient data obtained from the two largest trials of blood pressure lowering, the INTERACT2 [4] and the ATACH-II trials [5], demonstrated that achieving and maintaining a systolic blood pressure around 120–130 mmHg in the first 24 h is safe and might be associated with improved functional outcome [13].

Hematoma volume and location are the two main predictors of outcome related to the hematoma itself [11, 13, 14]. Hematomas greater than 30 ml are statistically associated with unfavorable outcome [15, 16]. The combination of hematoma volume greater than 60 ml with a GCS lower than 8 has a predicted 30-day mortality greater than 90% [16]. Acute hematomas greater than 150 mL usually leads to death due to the abrupt increase in intracranial pressure and consequently the reduction in cerebral perfusion pressure below critical levels [16].

Regardless hematoma volume, hemorrhages occurring in the posterior fossa (specially the cerebellum) may be life-threatening because the infra-tentorial space is smaller and less complacent than supratentorial area [17, 18]. Infra-tentorial hemorrhages could cause acute hydrocephalus due to fourth-ventricle compression and also lead to direct brainstem herniation [18]. Therefore, in posterior fossa hematoma evacuation may be considered as lifesaving option in patients with larger hematomas, brainstem compression, hydrocephalus, or clinical deterioration, though robust data is limited [17].

**Fig. 1:**
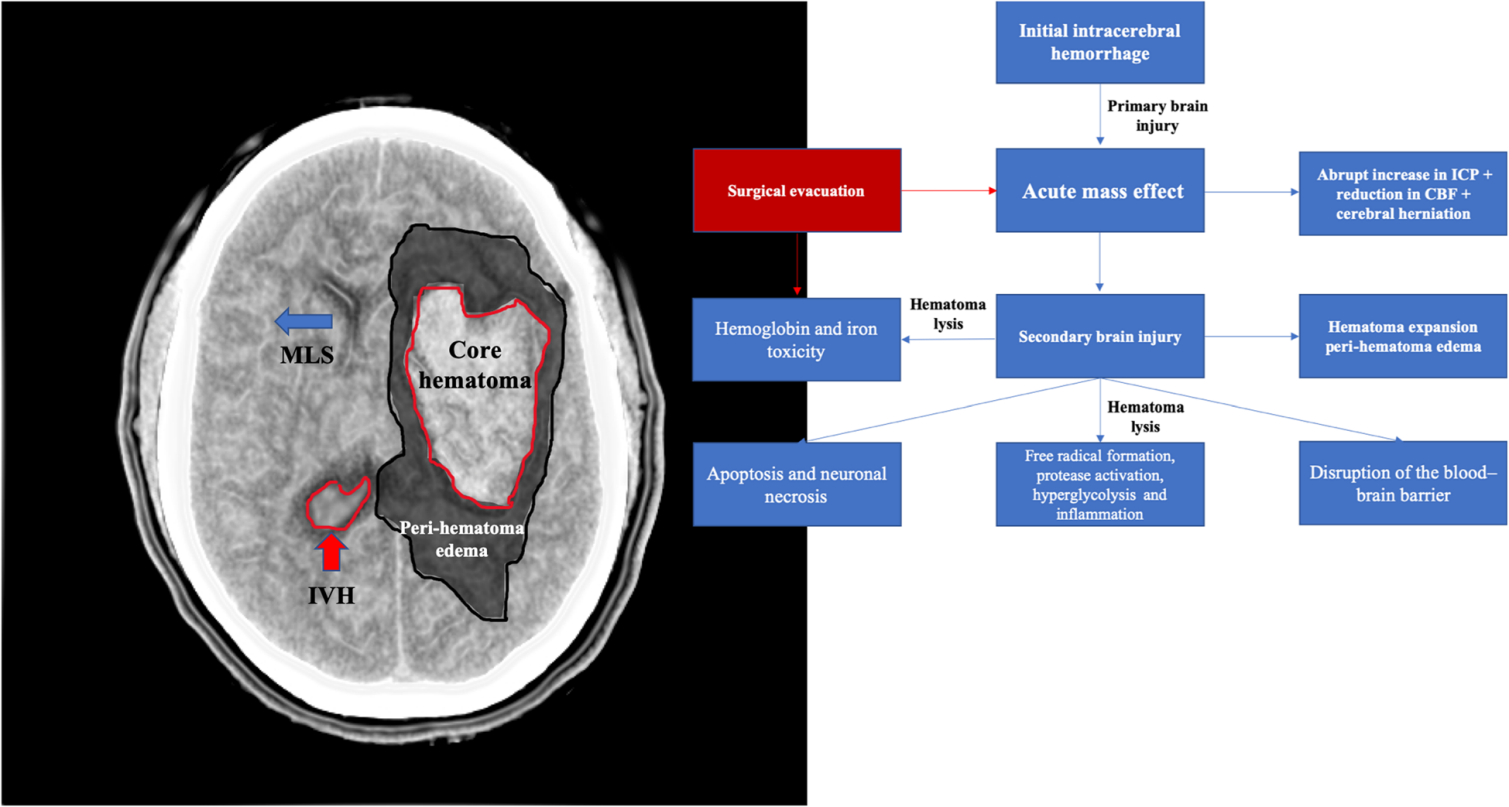
Mechanisms of secondary brain injury after ICH. MLS - midline shift; IVH - intraventricular hemorrhage

Additional to the physical effects of the initial and expanding hemorrhage, there are the effects of persistent hematoma and its blood products leading to a complex cascade of events (Fig. 1) [10, 19, 20].

The majority of ICH patients may not require surgery; however, there is a beneficial hypothesis for early surgical removal of an intraparenchymal hematoma. This benefit is based on the assumption that clot removal would restore the cerebral architecture, reducing mass effect and correcting or avoiding midline shift, and therefore it would improve cerebral perfusion by decreasing intracranial pressure. Additionally, hematoma drainage could prevent or at least reduce the cascade of secondary brain injury (Fig. 1) due to the deleterious effects of hemoglobin and its products into the brain. However, the surgical removal of a blood clot within the brain is not free of risks. In order to reach the hematoma that usually takes deep brain structures, a large layer of healthy cerebral tissue needs to be dissected, usually under general anesthesia. Additionally, postsurgical complications, such as hemorrhages and infections, are not uncommon in this clinical scenario, which increase the rates of mortality and unfavorable outcome [8, 21].

**Fig. 2:**
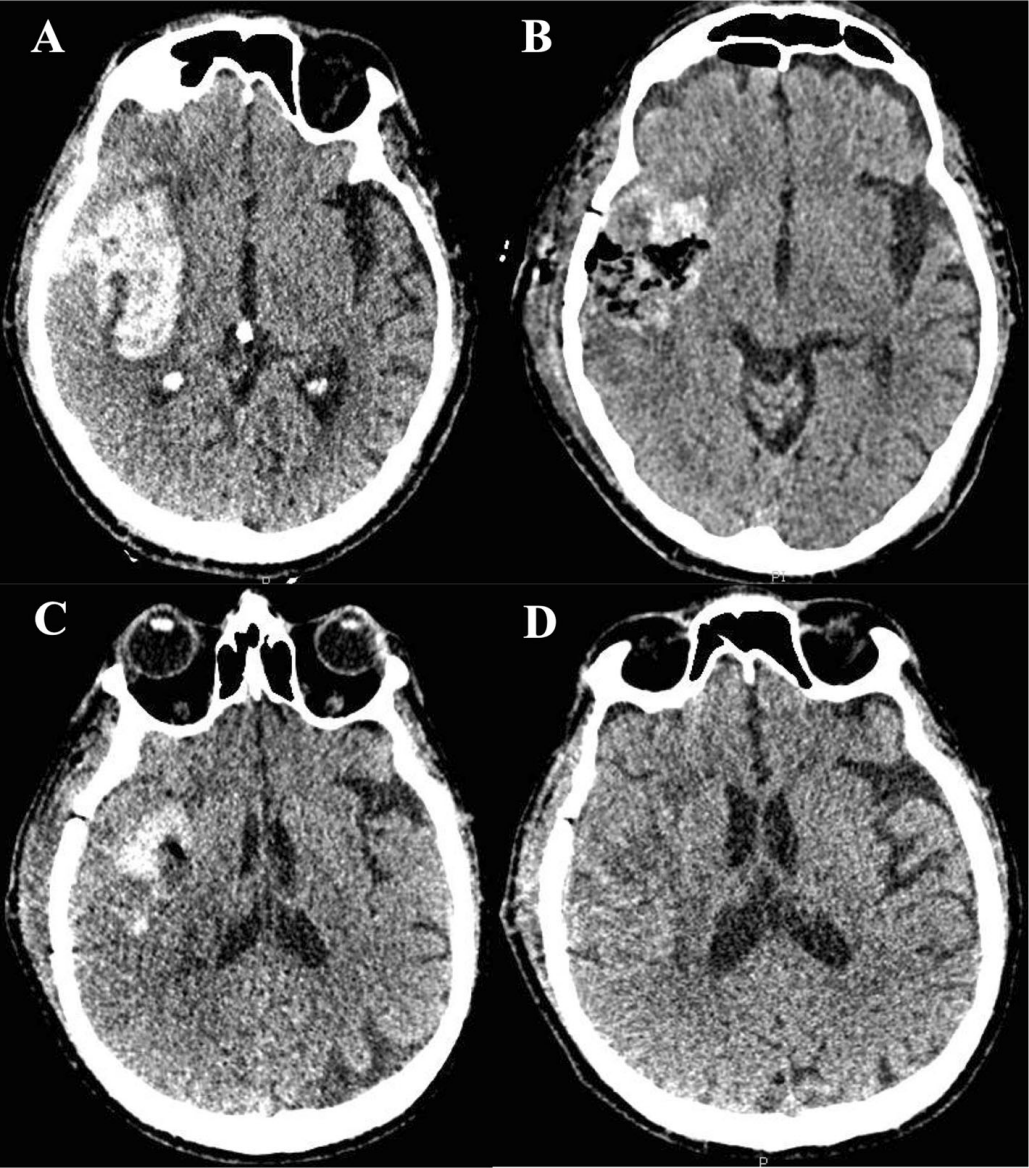
Case 01 of open craniotomy for hematoma drainage. **a** Day 1—a large intraparenchymal hematoma centered on the putamen, right insular, and frontotemporal region, with extravasation into the subarachnoid space of the sylvian fissure and temporal fossa, measuring about 6.1 × 4.5 × 4.8 cm on its largest axes. **b** Day 2—Hematoma was surgically removed by open craniotomy. CT shows signs of surgical manipulation characterized by enlargement and densification of soft tissue planes with gaseous foci underlying the right parietotemporal craniotomy. There was reduction of the dimensions of the intraparenchymal hematoma. **c** Day 7—Follow-up CT scan 6 days after surgical drainage. **d** Day 21—Follow-up CT scan 21 days after surgical drainage. Patient was discharged home after this last CT scan with a modified Rankin scale 4 (able to walk with assistance)

**Fig. 3:**
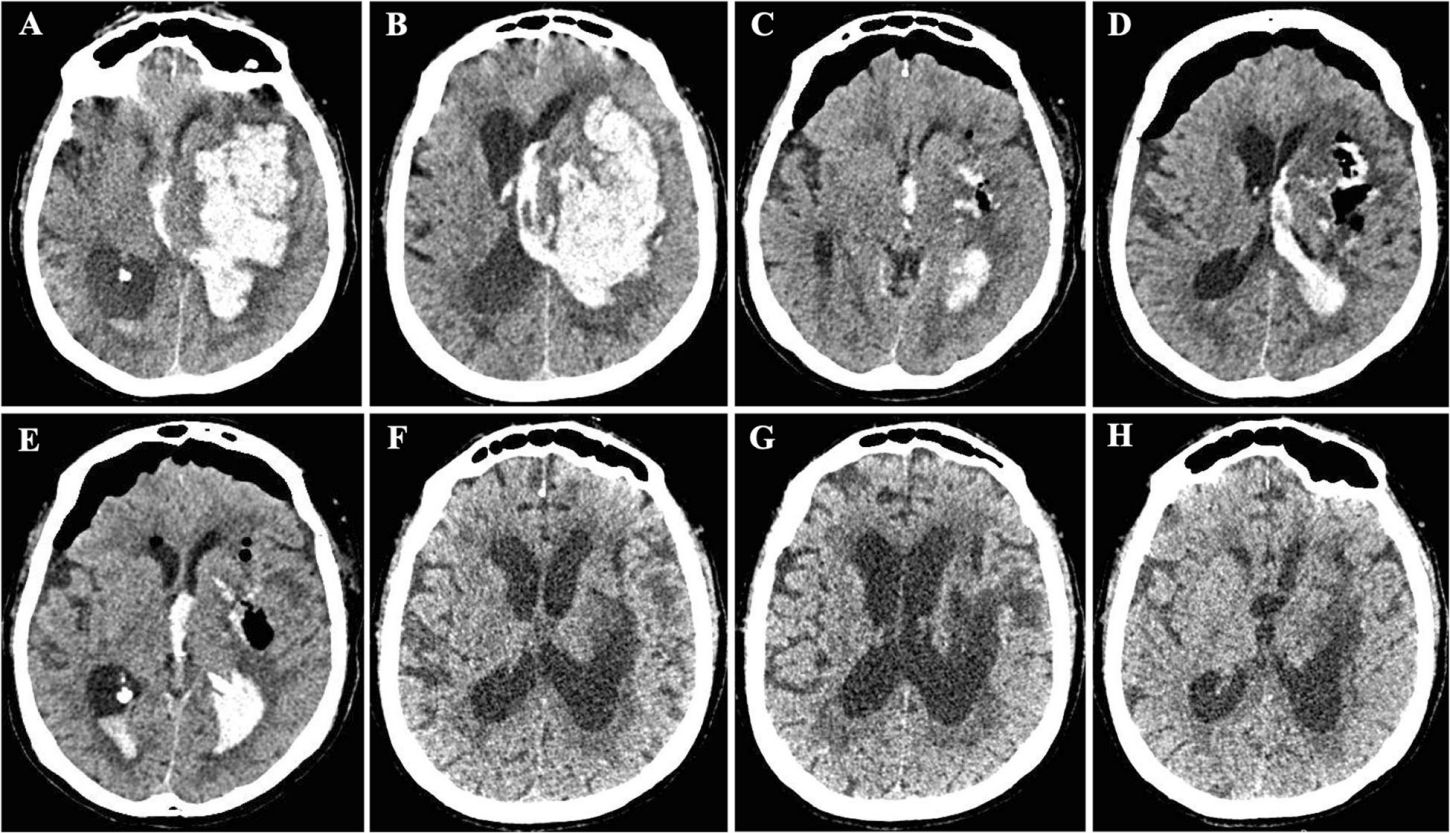
Case 02 of open craniotomy for hematoma drainage. **a**, **b** Day 1**—**Large hematoma in the left cerebral hemisphere leading to collapse of the left lateral ventricle with a midline shift of 12 mm, with a large left ventricular and third ventricle flooding, as well as diffuse effacement of cortical sulci of that hemisphere. **c**–**e** Day 2—Left frontoparietal craniotomy, with well-positioned bone fragment, aligned and fixed with metal clips. Reduction of the left frontal/frontotemporal intraparenchymal hematic content, with remnant hematic residues and air foci in this region. There was a significant reduction in the mass effect, with a decrease in lateral ventricular compression and a reduction in the midline shift. Bifrontal pneumocephalus causing shift and compressing the adjacent parenchyma. **f**–**h** Day 36—Resolution of residual hematic residues and pneumocephalus. Encephalomalacia in the left frontal/frontotemporal region. Despite the good surgical results, the patient remained in vegetative state

**Fig. 4:**
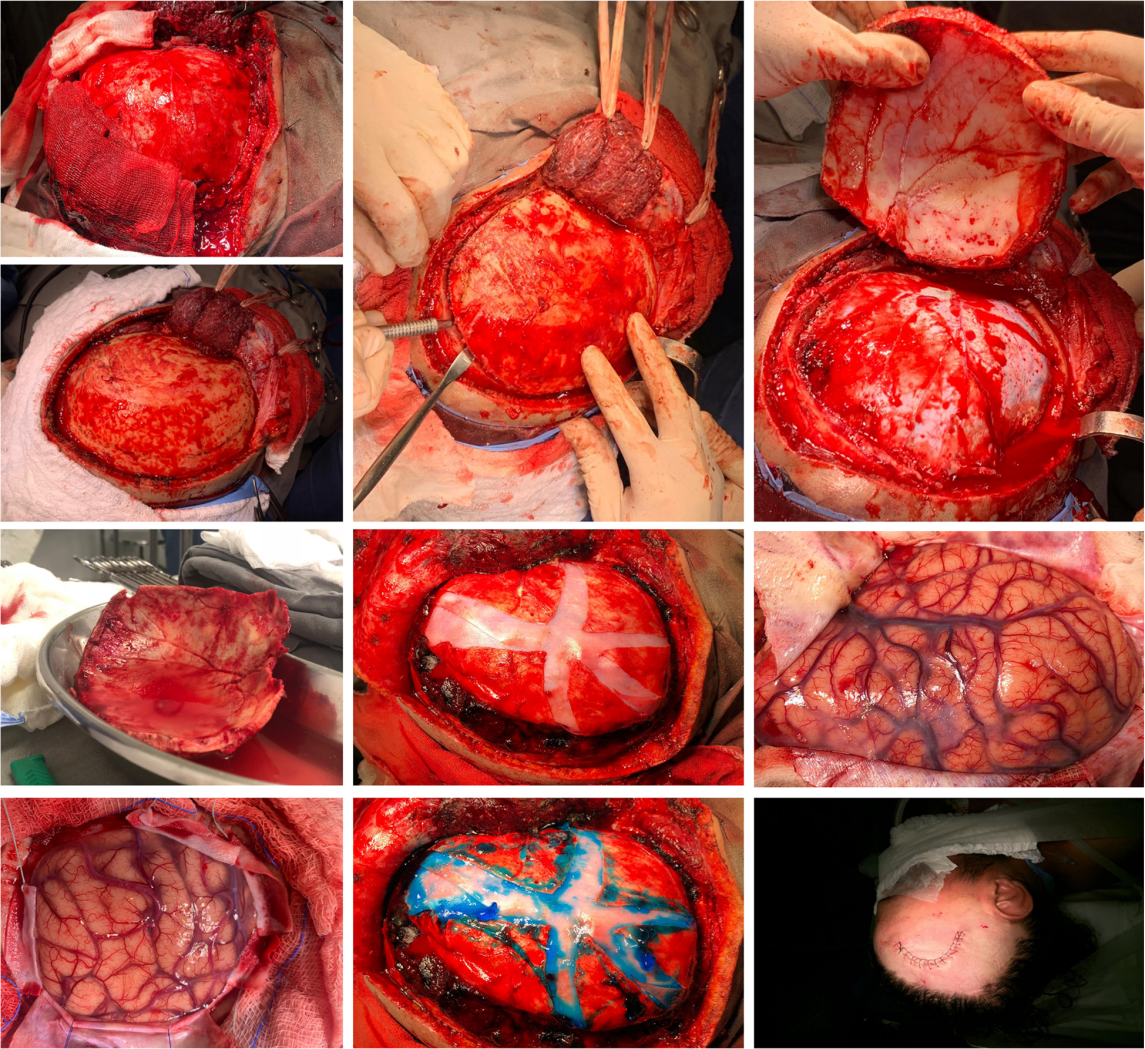
Open craniotomy. Patient lies on an operating table and receives general anesthesia. The head is set in a three-pin skull fixation device attached to the operating table, in order to hold the head standing still. Once the anesthesia and positioning are established, skin is prepared, cleaned with an antiseptic solution, and incised typically behind the hairline. Then, both skin and muscles are dissected and lifted off the skull. Once the bone is exposed, burr holes are built in by a special drill. The burr holes are made to permit the entrance of the craniotome. The craniotomy flap is lifted and removed, uncovering the dura mater. The bone flap is stored to be replaced at the end of the procedure. The dura mater is then opened to expose the brain parenchyma. Surgical retractors are used to open a passage to assess the hematoma. After the hematoma is drained, the retractors are removed, the dura mater is closed, and the bone flap is positioned, aligned, and fixed with metal clips. Finally, the skin is sutured

**Fig. 5:**
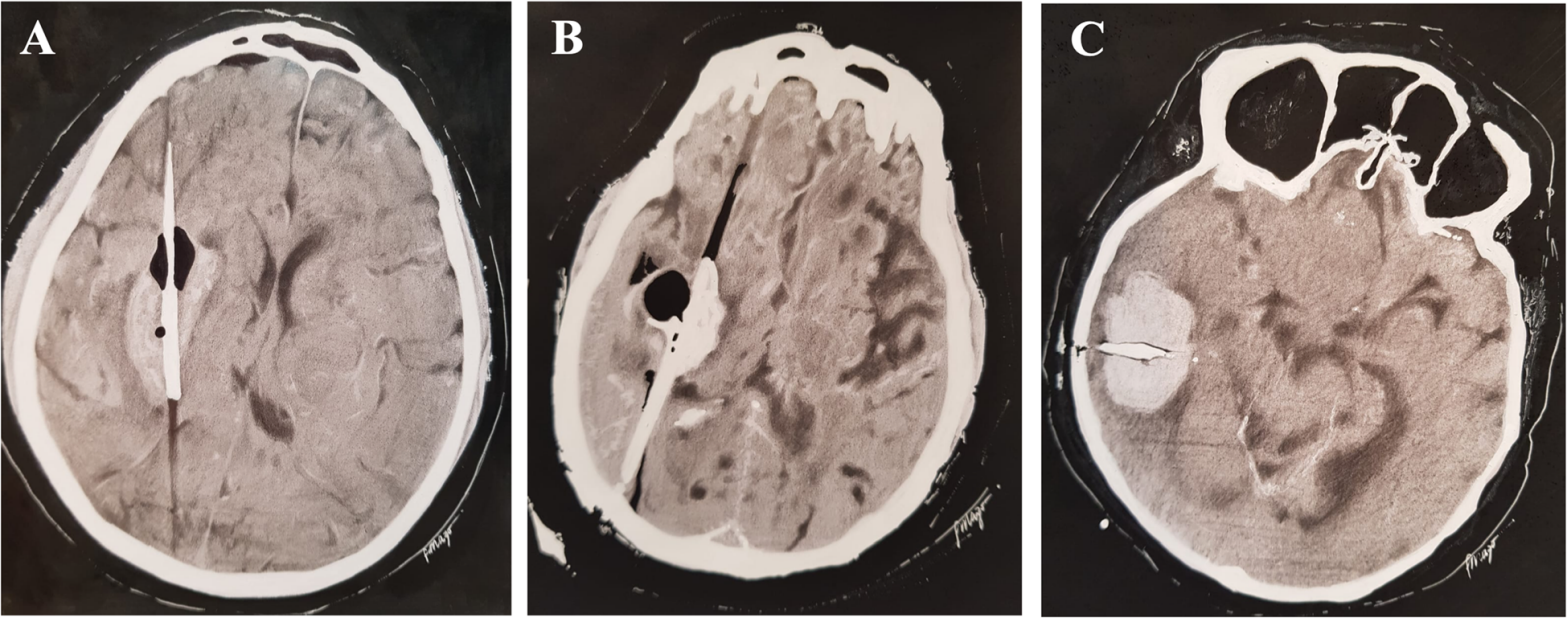
Surgical trajectories of catheter insertion in minimally invasive surgery. This figure was adapted from previously published images by Fam et al. [49]. **a** Basal ganglia hemorrhage (caudate, putamen, or anterior capsule)**.** The catheter is inserted through the forehead. Catheter trajectory: along the clot longitudinal axis. **b** Thalamic or posterior capsular hemorrhage. The catheter is inserted through the parietal-occipital area. Catheter trajectory: along the clot longitudinal axis. **c** Lobar hemorrhage. The catheter is inserted through the superficial area contiguous to the clot. Catheter trajectory: along the clot widest axis

Several surgical approaches exist, which include (a) the insertion of external ventricular drain (EVD) for intraventricular hemorrhage (IVH) management and intracranial pressure (ICP) monitoring, (b) craniotomy for hematoma drainage (Figs. 2, 3, and 4), (c) decompressive craniectomy with or without hematoma drainage, and lastly (d) the use of minimally invasive the use of minimally invasive approaches (Fig. 5).

### External ventricular drain insertion for intraventricular hemorrhage management

Intraventricular hemorrhage occurs in approximately 45% of patients with ICH, and it is an independent predictor of unfavorable outcome [22]. Intraventricular hemorrhage can interfere with the normal flow of cerebrospinal fluid, which may cause acute hydrocephalus, and in severe cases can lead to intracranial hypertension. Patients with acute hydrocephalus due to IVH or large intraparenchymal hematomas with mass effect associated with impaired level of consciousness (i.e., GCS ≤ 8) may require the urgent placement of an EVD, which allows for cerebrospinal fluid drainage and ICP monitoring [9, 23]. The goals for ICP and cerebral perfusion pressure (CPP) do not differ from those for traumatic brain injury, which suggests keeping an ICP < 20 mmHg (in more recent guideline 22 mmHg) and a CPP > 60 mmHg [23].

In severe cases, the large volume of blood in the ventricular system can cause drain malfunction and frequent catheter obstruction; therefore, the use of procedures to improve clot clearance have been tested. A phase III trial, the randomized, multicenter, multiregional, placebo-controlled CLEAR III trial [24] compared the use of low intraventricular dose (1 mg every 8 h, to a maximum of 12 doses) of recombinant tissue plasminogen activator (r-tPA) with placebo (i.e., normal saline) for patients with small spontaneous ICH (i.e., volume less than 30 ml) and an IVH obstructing the third or fourth ventricles.

The study investigators were solicited to clear as much IVH as possible, until the third and fourth ventricles were opened; or the IVH mass effect was relieved; or 80% of intraventricular clot was removed; to a maximum of 12 r-tPA doses. The intraventricular clot volumes were analyzed by a core laboratory using semi-automated segmentation and Hounsfield thresholds.

Five hundred participants, who routinely received an EVD, were included from 73 sites between 2009 and 2014. The primary favorable outcome defined as a 6-month modified Rankin scale (mRS) of 0–3 was not significantly different between the r-tPA and saline groups [r-tPA group 48% vs saline 45%; risk ratio (RR) 1.06 (95% CI 0.88–1.28; *p* = 0.554)]. The treatment with r-tPA was associated with 11% lower case fatality [46 (18%) vs saline 73 (29%), hazard ratio 0.60 (95% CI 0.41–0.86), *p* = 0.006), to the cost of an 8% increase in the proportion of patients in a vegetative state (i.e., mRS = 5); [42 (17%) vs 21 (9%); RR 1.99 (95% CI 1.22–3.26), *p* = 0.007]. Complications such as ventriculitis, symptomatic hemorrhage, and serious adverse events were not higher in the r-tPA group.

Eighty-two patients (33%) in the treatment group vs 24 patients (10%) in the control group achieved the endpoint of 80% intraventricular clot removal. A pre-specified secondary analysis showed a significant relation between the amount of clot removed [per clot remaining (mL) as measured by normalized AUC] and both mRS ≤3 [adjusted OR 0.96 (95% CI 0.94–0.97); *p* < 0.0001], and case fatality [adjusted HR of death per mL of time-weighted clot volume remaining 1.03 (95% CI 1.02–1.04); *p* < 0.0001]. One of the reasons why treatment was not effective may be explained by the fact that only one third of patients in the intervention group achieved the goal of clot removal.

Therefore, despite the association between the amount of clot removal and improved chances of mRS ≤ 3 (secondary analysis), the use of intraventricular r-tPA in patients with IVH obstructing the third or fourth ventricles did not improve 6-month functional outcome (primary outcome) when compared with placebo [24], and might increase the rates of survivorship with severe disability [25].

The use of dual EVD insertion, with and without thrombolytic therapy [26], and the combination of intraventricular fibrinolysis with lumbar drainage [27] have also been tested. The first was shown to increase clot resolution for large IVH (> 40 ml), with and without thrombolytic therapy [26]. The second significantly reduced the shunt dependency for hydrocephalus after IVH [27].

Another possible approach to manage IVH secondary to spontaneous intracerebral hemorrhage is the clot removal by neuroendoscopy in combination with EVD placement. Neuroendoscopy is minimally invasive and has high rates of clot evacuation with small proportions of surgical complications. A meta-analysis of 11 studies, which included only 5 randomized clinical trials, found the neuroendoscopy + EVD was superior than the EVD + r-tPA approach in terms of mortality, effectiveness of IVH evacuation, favorable functional outcome, and also the need for ventriculoperitoneal shunt [27, 28]. However, despite these interesting preliminary results, the efficacy of neuroendoscopic + EVD insertion for the treatment of IVH remains unclear [9]. Additionally, no definitive evidence concerning the preference between neuroendoscopy vs. EVD alone to treat IVH exists, because of limited data published to date [28].

### Craniotomy for supratentorial hemorrhage drainage

Although the role of open surgery to treat patients with spontaneous ICH remains controversial, the use of craniotomy for supratentorial hematoma drainage is the most common strategy applied in most centers and also the most studied approach so far (Figs. 2, 3, and 4) [29, 30].

The first controlled study dated from the early 1960s [31], when McKissock and colleagues reported a prospective controlled trial of 180 patients randomized to craniotomy for hematoma evacuation vs. conservative management. Forty-six (51%) patients in the conservative group vs. 58 (65%) patients in the surgical group died. The authors were “unable to demonstrate any benefit from surgery in regard either to mortality or morbidity” [31]. Additionally, patients who were hypertensive had their mortality rate increased by surgery compared with conservative management. It is important to mention that since this early study, conservative management did not mean withholding life support. McKissock and colleagues stated “we would stress that conservative treatment involves more than ‘doing nothing’; nursing care of a high standard, constant medical supervision, and control of cerebral edema and pulmonary complications are implicit in the term” [31].

Decades have passed, but the role of craniotomy for hematoma evacuation remains a topic of hot debate, despite the publication of numerous studies (Table 2) [32–48], including two well-designed, well-powered (10% absolute increase in favorable outcome in the surgical group), multicenter, multinational, randomized clinical trials [40, 44].

The Surgical Trial in Intracerebral Hemorrhage (STICH) [40] was the first well-powered, multicenter, multinational, randomized clinical trial to compare the benefits of early hematoma drainage with initial conservative management. One thousand and thirty-three (1033) patients with lobar or ganglionic spontaneous supratentorial hematoma were enrolled from 83 centers in 27 countries, to undergo early hematoma evacuation (within 24 h of randomization and within 72 h of ictus) or conservative management (i.e., best medical care with delayed surgery if necessary). Delayed hematoma evacuation was allowed in the conservative group if necessary, in case of delayed neurological worsening.

The *study inclusion criteria* included the following: (a) confirmation of a spontaneous supratentorial intracerebral hemorrhage by noncontrast CT head performed within 72 h of initial symptoms; (b) a hematoma diameter ≥ 2 cm; (c) GCS ≥ 5; and finally (d) clinical uncertainty, i.e., the responsible neurosurgeon was unsure about the clinical benefits of either treatment. *Exclusion criteria* included the following: (a) hemorrhage due to a vascular abnormality (e.g., cerebral aneurysm or an arteriovenous malformation); (b) hemorrhage due to tumors or trauma; (c) posterior fossa hemorrhage (i.e., cerebellar hemorrhage or supratentorial hemorrhage extending into the brainstem); (d) if the surgery could not be performed within 24 h of randomization; (e) if the patient was physically or mentally disabled before hemorrhage.

The primary outcome was death or disability according to the extended Glasgow outcome scale (eGOS—Table 2) assessed by structured postal questionnaires at 6 months and evaluated by blinded intention to treat analyses. The authors divided the patients in two groups of estimated prognoses (good and poor) according to the following equation:

$$ \mathrm{Prognostic}\ \mathrm{score}=\left(10\times \mathrm{admission}\ \mathrm{Glasgow}\ \mathrm{coma}\ \mathrm{score}\right)-\mathrm{age}\ \left(\mathrm{years}\right)-\left[0.64\times \mathrm{volume}\ \left(\mathrm{ml}\right)\right] $$

A score > 27.672 was used as a cutoff point for a good prognosis. Therefore, patients predicted of poor outcome according to the above described prognosis-based methodology, a favorable prognosis was considered if eGOS = 4–8 was achieved, while for those patients with a predicted good outcome, a favorable outcome included eGOS = 5–8.

At 6 months, 51 patients (5%) had been lost to follow-up. No overall benefit in functional outcome was found with early hematoma drainage, since 122 (26%) patients progressed to a favorable outcome in the surgical group vs. 118 (24%) patients in the initial conservative treatment group (odds ratio 0·89 [95% CI 0·66–1·19], *p* = 0·414) [40]. Additionally, mortality rate was similar in both groups [36% surgery vs. 37% conservative; OR 0.95 (0.73–1.23), *p* = 0.707].

Several prespecified subgroup were analyzed by intention to treat, which included (a) age (< 65 vs ≥ 65 years); (b) hematoma volume (< 50 ml vs ≥ 50 ml); (c) GCS (≤ 8 vs 9 to 12 vs ≥ 13); (d) hemorrhage location (lobar vs basal ganglia/thalamic hematoma, or both); (e) anticoagulation or thrombolytic-associated hemorrhage; (f) severity of neurological deficit; (g) type of intended operation (craniotomy vs other); (h) the hematoma side (left vs right); (i) the depth from the cortical surface (< 1 cm vs ≥ 1 cm); and lastly (j) country. There is no benefit of early surgery across all prespecified subgroups, except for a signal of possible benefit in the subgroup of patients with superficial hematomas (absolute benefit 8%; 0–15), with a significant interaction between hematoma depth and surgery (*p* = 0.02).

Consequently, a second study was performed by the same group of investigators to test the hypothesis that patients with superficial hematomas within 1 cm from cortical surface could benefit from early hematoma removal (early surgery versus initial conservative treatment in patients with spontaneous supratentorial lobar intracerebral haematomas - STICH II) [44]. The study was also an international, multicenter, prospective, randomized trial, which included only patients with superficial hematomas within 1 cm from the cortical surface of the brain. Patients with IVH, hematoma < 10 ml or > 100 ml, comatose patients (i.e., motor GCS < 5 and eye GCS < 2 at randomization), and patients admitted beyond 48 h of ictus were excluded. The same strategy to assess and dichotomized the primary outcome described above was used (i.e., death or disability by the extended Glasgow outcome scale assessed by structured postal questionnaires at 6 months and evaluated according to the prognosis-based outcome).

A total of 601 patients were included from 78 centers in 27 countries (307 in the early surgery and 294 in the conservative group), with an excellent follow-up at 6 months [589 out of 601 (98.0%) patients were available for follow-up at 6 months]. Nor overall benefit in functional outcome [62% unfavorable outcome in the surgical group vs. 59% in the initial conservative treatment group (absolute difference 3.7% (95% CI − 4.3 to 11.6), odds ratio 0.86 (0.62 to 1.20); *p* = 0.367)], neither mortality benefit was detected [18% in the surgical group vs. 24% in the conservative group (OR 0.71, 95% CI 0.48 to 1.06; *p* = 0.095)].

When the STICH trials results are combined in a meta-analysis with other 13 studies (sample size of 3366) [31–36, 38–41, 44], patients with predicted poorer prognosis, delayed clinical deterioration, or superficial lobar ICH without IVH may have a potential survival benefit [OR 0.74 (95% CI 0.64–0.86; *p* < 0.0001)] [44]. However, there is a substantial heterogeneity in the quality of studies (*p* = 0.0002), since the trials included have different patients’ populations and used multiple surgical strategies (e.g., craniotomy, endoscopic surgery, stereotactic ± plasminogen activator), limiting the validity of these results.

In summary, the two largest well designed, well-powered randomized clinical trials comparing early hematoma evacuation by craniotomy vs. initial conservative management did not show functional outcome or mortality benefit with early hematoma evacuation (Tables 1 and 2). Early craniotomy for hematoma evacuation cannot be recommended as routine care for patients suffering from supratentorial ICH, especially in deep hemorrhages and in small lobar hemorrhages with preserved level of consciousness. However, craniotomy for hematoma drainage is an important life-saving measure in critical situations, such as large hematomas with mass effect and midline shift leading to altered level of consciousness or when delayed neurological deterioration occurs due to hematoma expansion [21]. The ideal patients who would benefit from early surgery is still to be determined.

**Table 1:** Pros and cons in the STICH trials

Strong points of STICH trials • Well-designed, well-powered randomized clinical trials • Multicenter, multinational • The research question tested was biologically plausible • Very low rate of missing long-term follow-up • Adoption of prognosis-based outcome • The surgical group was limited to patients who had early surgery (within 72 h of hemorrhage and within 24 h of randomization) • Although the patients, surrogates, and site investigators were aware of treatments’ allocation; the data manager was the only study person that knew patients’ allocation at the coordinating center.	
Weak points of STICH trials • Large cross-over from conservative to surgical group: approximately one quarter of patients in the initial conservative group crossed over to surgery due to delayed neurological deterioration. These patients were more likely to bear hematomas ≥ 50 ml, and those with a predicted poor prognosis • The clinical uncertain principle: patients were only included if the responsible neurosurgeon was unsure about the clinical benefits of either treatment. Therefore, patients who were considered to benefit from hematoma evacuation were not included in the study. The evaluation and decision were on discretion of responsible neurosurgeon, leading to selection bias. However, including comatose patients with expanding hematomas or brain herniation in the conservative management would not be ethically acceptable, since surgery is likely a life-saving measure for this subset of patients. • If no patient had crossed over to surgery, the rates of unfavorable outcome and death in the initial conservative management group may have been higher. • Large number of excluded patients in the STICH II trial (> 3300) because of impaired level of consciousness at the time of randomization, which adds additional selection bias to the study. Patients with preserved level of consciousness are those with less severe hemorrhages, therefore these patients have a higher likelihood of favorable outcome, irrespective of treatment.

**Table 2:** Randomized controlled trials comparing hematoma evacuation vs. conservative medical management or placebo

Study	Design	Surgical technique	Included patients (conservative/surgery)	Crossover	Timing of surgery (h)	Outcome definition and timing of assessment	Findings
McKissock et al. (1961) [31]	Double-center, prospective randomized clinical trial	Craniotomy	180 (91/89)	1 patient	72 (most patients were treated within 48 h)	- Full work; partial disability; total disability - 6 months	The authors were “unable to demonstrate any benefit from surgery in regard either to mortality or morbidity”
Auer et al. (1989) [32]	Prospective single-center randomized clinical trial	Endoscopic-guided evacuation	100 (50/50)	Not reported	48	- 6 Grades Outcome* - 6 months	Lower mortality (30 vs. 70%, *p* < 0.05) and higher rates of favorable outcome (40 vs. 25%, *p* < 0.01) in the surgical group. These results were limited to patients with subcortical hemorrhages, who were alert or somnolent perioperatively. The outcome was not improved by surgery in putaminal or thalamic hemorrhages.
Juvela et al. (1989) [33]	Prospective single-center randomized clinical trial	Craniotomy	52 (26/26)	Not reported	48	- Glasgow Outcome Scale - 1, 6, and 12 months	Surgery did not offer any advantage over conservative treatment. Additionally, in comatose patients (GCS = 7–10), surgery decreased mortality, but patients survived with poor quality of life.
Batjer et al. (1990) [34]	Prospective single-center randomized clinical trial	Craniotomy	- Total = 21 - 9 best medical management - 4 best medical management + intracranial pressure monitoring - 8 surgical evacuation	None	24	1 (dead or vegetative) 2 (dependent) 3 (independent at home) 4 (return to prestrike activity) - 6 months	- 4 out of 8 patients died in the surgical group - 2 were capable of independent life. - Surgery was ineffective compared with best medical management or best medical management + ICP monitoring
Morgenstern et al. (1998) [35]	Prospective single-center randomized clinical trial	Craniotomy	41 (34/7)	None	12	- Barthel score and mortality - 1 and 6 months	- 6-month mortality rate and functional outcome favor conservative group
Zuccarello M et al. (1999) [36]	Prospective three centers randomized clinical trial	Craniotomy CT-guided stereotaxic placement of a catheter	20 (11/9) - 5 craniotomy - 4 stereotaxic evacuation - 11 best medical therapy	1 patient	24	- Glasgow Outcome Scale - 3 months	No difference in the likelihood of a good outcome or mortality at 3 months.
Morgenstern et al. (2001) [37]	Prospective single-center cohort	Ultra-early craniotomy	11 (all surgical)	None	4	- Barthel score, modified Rankin Scale and mortality - 6 months	- Interrupted after planned interim analysis, because of increased rates of rebleeding with ultra-early craniotomy
Teernstra et al. (2003) [38]	Multicenter randomized controlled trial (13 centers)	Stereotactic aspiration combined with urokinase injection	71 (35/36)	- One crossover from medical to craniotomy - One patient from stereotactic group underwent craniotomy - Four patients received no stereotactic drainage	72	- Death - 6 months	- The trial was prematurely stopped due to slow recruitment - Mortality 3-months was not statistically significant, 56% in the surgical group vs. 59% in the nonsurgical group
Hattori et al. (2004) [39]	Prospective single-center randomized clinical trial	Stereotactic evacuation	242 (121/121)	None		- Modified Rankin Scale - 12 months	Stereotactic evacuation reduced mortality and improved functional outcomes in patients with neurological Grade 3 (eyes closed but open to strong stimuli)
STICH (2005) [40]	Multicenter randomized controlled trial (83 centers in 27 countries)	Craniotomy 75% Burrhole 8% Endoscopy 7% Stereotaxy 7% Other 3% Not recorded	1033 (530/503)	26%	72 h of ictus and 24 h of randomization	- Extended Glasgow Outcome Scale according to a prognosis-based methodology - 6 months	No overall benefit in mortality or functional outcome mortality was found with surgery
Pantazis et al. (2006) [41]	Prospective single-center randomized clinical trial	Craniotomy	108 (54/54)	2 patients	8	- Glasgow Outcome Scale - 12 months	- 33% of patients in the surgical group vs. only 9% of patients in the conservative group had a Glasgow Outcome Scale > 3 (*p* < 0.05). - There is no mortality benefit with surgery. - When functional outcome was stratified by neurological status on admission, hematoma volume and location, no benefit with surgery was seen for patients with GCS < 8 or ICH ≥ 80 ml at enrollment
Kim et al. (2009) [42]	Prospective single-center randomized clinical trial	Stereotactic-guided evacuation	387 (183/204)	23 patients (they were excluded from the analysis)	12 h up to 5 days	- Modified Barthel Indices (MBI) and the modified Rankin Scale - 6 months	- Stereotactic-guided evacuation had a significant effect on a functional recovery - MBI was 90.9 in the surgical group vs. 62.4 in conservative group (*p* < 0.05) - mRS was 1.2 in the surgical group vs. 3.0 in the conservative group (*p* < 0.05)
Wang et al. (2009) [43]	Multicenter randomized controlled trial (42 centers)	Minimally invasive craniopuncture combined with urokinase injection	377 (182/195)	- 16 patients (7 patients randomized to craniopuncture therapy refused operation, and 9 patients randomized to conservative treatment crossover to surgery) - they were excluded from the analysis	Mean time in hours from stroke onset to operation (SD) = 21.1 (15.9)	- Modified Rankin Scale and Barthel Index - 3 months	- No significant difference in activities of daily living score - The proportion of dependent survival patients (modified Rankin scale > 2) in the craniopuncture group (40.9%) was significantly lower than that in the conservative group (63.0%) - No significant difference in the cumulative fatality rates
STICH II (2013) [44]	Multicenter randomized controlled trial (78 centers)	Craniotomy 99% Craniectomy < 1% †Minimally invasive 1%	601 (294/307)	21%	48 h of ictus and 12 h of randomization	- Extended Glasgow Outcome Scale according to a prognosis-based methodology - 6 months	- 59% of the surgery group had an unfavorable outcome versus 62% in the initial conservative group (absolute difference = 3.7%, 95% CI = − 4.3–11.6, OR = 0.86, 95% CI = 0.62–1.20, *p* = 0.367). - No overall benefit in functional outcome or mortality was found
MISTIE (2016) [45]	Randomized, controlled, open-label, phase 2 trial (26 centers)	Image-guided, catheter-based, stereotactic aspiration and thrombolysis (alteplase 0.3 mg or 1.0 mg every 8 h for up to nine doses)	96 (42/54)	- 4 patients in the conservative group and 2 in the minimally invasive surgery plus alteplase underwent craniotomy	- 57% underwent surgery within 36 h, while 43% underwent surgery beyond 36 h	- Primary outcomes: 30-day mortality; 7-day procedure-related mortality; 30-day bacterial brain infection; symptomatic bleeding within 72 h after the last dose.	- Primary outcomes did not differ between the two groups - Asymptomatic hemorrhages were more common in the surgical group
Intraoperative Stereotactic Computed Tomography-Guided Endoscopic Surgery for Brain Hemorrhage (2016) [46]	Multicenter randomized controlled trial (78 centers)	Intraoperative Stereotactic Computed Tomography-Guided Endoscopic Surgery	24	None	48	- Modified Rankin Scale - 6 and 12 months	- Early computerized tomographic image-guided endoscopic surgery is a safe and effective - One bleed occurred peri-operatively - The surgical intervention group had a greater percentage of patients with favorable neurological outcome
CLEAR III (2017) [24]	Randomized, multicenter, multiregional, placebo-controlled trial (73 centers)	Thrombolytic removal of intraventricular hemorrhage (alteplase 1 mg through an EVD, every 8 h, to a max. 12 doses)	500 (251 placebo/ 249 alteplase)	None	72	- Modified Rankin Scale - 6 months	- The injection of intrathecal alteplase did not improve functional outcomes in IVH patients - 6-month modified Rankin scale (mRS) of 0–3 was not significantly different between the r-tPA and saline groups - Treatment was associated with lower case, to the cost of a higher proportion of patients in a vegetative state
MISTIE III (2019) [47]	Randomized, controlled, open-label, blinded endpoint phase 3 trial (78 centers)	Minimally invasive catheter evacuation followed by thrombolysis (alteplase 1.0 mg every 8 h for up to nine doses)	506 (251/255)	None	72	- Modified Rankin Scale - 12 months	- MISTIE did not improve long-term outcome - 110 patients (45%) in the MISTIE group vs. 100 patients (41%) in the conservative group achieved a favorable outcome [adjusted risk difference 4% (95% CI − 4 to 12); *p* = 0.33] - Meta-analysis including only multisite trials of MISTIE was performed by the authors and no significant benefit of MISTIE was found (OR 0.61, 95% CI 0.29–1.26).

†Minimally invasive procedures were burrhole with endoscopic evacuation in two patients and keyhole evacuation in one patient. *Grade 1—patient leads a full and independent life without neurological deficit; Grade 2—patient leads a full and independent life with minimal neurological deficit; Grade 3—patient has neurological or intellectual impairment, but is independent and able to work part-time; Grade 4—patient has neurological deficit, is unable to work but is capable of self-care; Grade 5—conscious patient totally dependent on others for activities of the day; Grade 6—dead

### Minimally invasive surgical approaches for ICH

The practice of open craniotomy is not without risks and complications, because it requires a large bone flap, the exposition of the brain tissue, which is dissected, retracted, and manipulated in order to reach the location of hemorrhage (Fig. 4). Healthy brain tissue is damaged during this process. Instead, the application of alternative approaches has been tested in this clinical scenario, which includes the use of minimally invasive techniques, which has the theoretical benefit of producing minimum surgical trauma to the normal brain tissue manipulated throughout the process of hematoma drainage (Fig. 5).

The first controlled trial of minimally invasive surgery for ICH was performed in the 1980s and compared the use of endoscopic hematoma evacuation with conservative management [32]. In neuroendoscopy, an endoscope that measures approximately 5 to 8 mm in diameter with a miniature high-definition video camera attached is introduced through a burr hole created in the skull. The neuroendoscope navigates across normal brain tissue into the hemorrhage. Once the hematoma is reached, the blood clot can be aspirated by the endoscope ± the assistance of fluid or thrombolytic irrigation. Auer et al. [32] were the first to report a study that included 100 spontaneous ICH patients, who presented with focal deficits ± altered level of consciousness; cerebral hematoma ≥ 10 ml, and who were treated within 48 h of ictus. The authors used a rigid 6-mm endoscope tube, which was utilized to continuously rinsed the hematoma cavity with what the authors described as “artificial cerebrospinal fluid at body temperature through one channel at a pressure between 10 and 15 mmHg” [32]. Then, at regular time intervals, the mixture of blood and artificial CSF was suctioned through a separate endoscope channel.

Outcome assessment was performed 6 months after hemorrhage by a scale similar to the modified Rankin scale. Lower mortality (42 vs. 70%, *p* < 0.01) and higher rates of favorable outcome (40 vs. 25%, *p* < 0.01) were achieved by the surgical group; however, these results were limited to patients with subcortical hemorrhages, who were alert or somnolent perioperatively. The outcome was not improved by surgery in stuporous or comatose patients, neither in putaminal or thalamic hemorrhages. Although these promising results were achieved by a first-generation endoscope without CT guidance, they still need to be replicated in a well-powered randomized clinical trial.

More recently, Dr. Vespa and colleagues published the ICES trial (Intraoperative Computed Tomography–guided Endoscopic Surgery for Brain Hemorrhage) [46], a pilot multicenter randomized controlled trial funded by the National Institutes of Neurological Disorders and Stroke, which tested the safety and effectiveness of CT-guided endoscopic drainage of ICH. The trial included adult patients with supratentorial ICH within 48 h of ictus, who had a hematoma volume > 20 ml + GCS > 5 + NIHSS > 5. Fourteen patients underwent intraoperative computerized tomographic image–guided endoscopic surgery, which resulted in instant decrease of hematoma volume by 68 ± 21.6% (interquartile range 59–84.5), within 29 h hemorrhage ictus. The surgical procedures were very quick [1.9 h (interquartile range 1.5–2.2 h)], with only one surgical complication described (i.e., a peri-operative surgical bleed). Compared with the medical group from the MISTIE trial [47], the surgical group had a non-significant higher rate of favorable neurological outcome by mRS at 12 months (42.9% versus 23.7%; *p* = 0.19); however, the study was not powered to assess functional outcome and mortality.

Additional to neuroendoscopy, a second mode of minimally invasive surgery for ICH is the stereotactic or image-guided placement of a catheter inside the hematoma, followed by the intra-hemorrhage thrombolysis, with the ultimate goal of improving hematoma lysis and drainage. Usually, a catheter is left in place inside the hematoma, where frequent small amounts of a recombinant tissue-type plasminogen activator (r-TPa) are delivered in order to drain the clot over a period of days.

The minimally invasive catheter evacuation followed by thrombolysis (MISTIE) technique can be technically challenging for surgeons of variable levels of experience. A phase II study has demonstrated the importance of ideal catheter placement in order to achieve optimal hematoma evacuation [45]. In order to guarantee the accuracy of catheter insertion and the efficacy of hematoma drainage, studies using the MISTIE technique need to unify protocols of neurosurgeon training about the best selection of multiple surgical trajectories for catheter insertion in different hematoma locations (Fig. 5) [49].

This minimally invasive technique seems to be safe [47], feasible, efficacious [49], and reproducible [49, 50], and it is also associated with reduction in hematoma volume and peri-hematoma edema [51]. However, recent data arising from randomized controlled trials did not shown significant benefit of this technique when compared with conservative management [47].

The phase 2 MISTIE study was a randomized, controlled, open-label, phase 2 trial performed in 26 centers across North America and Europe [45]. Adult patients with spontaneous ICH + hematomas volume ≥ 20 ml were randomly allocated to conservative management or MISTIE + alteplase protocol (0.3 mg or 1.0 mg every 8 h for up to nine doses). According to the study protocol, neurosurgeons had to follow a 10-step procedure, with the ultimate goal to achieve a decrease in clot size to less than 15 ml. A rigid cannula was inserted through a burr hole, followed by clot aspiration through a 10-ml syringe. The procedure was stopped when a resistance was felt. Thereafter, the rigid cannula was replaced by a tunneled soft catheter under image guidance, with positioning confirmed by follow-up CT scan. After at least 6 h of catheter placement, the administration of alteplase in a dose of 0.3 mg or 1.0 mg diluted in 1 ml of saline was performed, followed by 3 ml of flush, every 8 h. The catheter was locked by an hour after alteplase infusion. Thrombolytic administration was stopped when residual hematoma was ≤ 15 ml, or when the maximum nine doses of alteplase were given, or in case of hemorrhagic complication, defined as a persistent decrease ≥ 2 points on the motor component of GCS, associated with an increase in the hematoma volume confirmed by CT scan. A total of 96 patients were included (54 in the intervention group and 42 in the conservative group). Thirty-day mortality [9.5%, (95% CI 2.7–22.6) vs. 14.8%, (6.6–27.1), *p* = 0.542], symptomatic bleeding [2.4%, (0.1–12.6) vs. 9.3%, (3.1–20.3), *p* = 0.226], and cerebral infections [2.4%, (0.1–12.6) vs. 0%, (0–6.6), *p* = 0.438] were not different between intervention and conservative groups, respectively. Only asymptomatic hemorrhage was more frequent in the intervention group [22.2%; (95% CI 12.0–35.6) vs. 7.1%; (1.5–19.5); *p* = 0.051) [45].

This pilot phase 2 study showed that intracerebral hemorrhage can be aimed and drained safely using serial thrombolytic injections through a stereotactically targeted catheter; therefore, a phase 3 trial was carried out.

The MISTIE III trial was an open label, phase 3 trial carried out at 78 hospitals in North America, Europe, Australia, and Asia [47]. The procedure for catheter placement and alteplase injection followed the same steps described above, except the dose of alteplase dose that was limited to 1.0 mg every 8 h to a maximum of nine doses. Adult patients with spontaneous supratentorial ICH + hematoma volume ≥ 30 ml + GCS ≤ 14 or NIHSS ≥ 6, and hematoma stability (hematoma expansion < 5 ml) for at least 6 h after diagnostic CT scan. A total of 506 patients were randomized (255 to MISTIE group vs. 251 to conservative management). The primary outcome was the percentage of patients with favorable functional outcome according to the mRS (0 to 3) at 12 months. The primary outcome was adjusted to ICH stability size, age, GCS, stability IVH size, and hematoma location. Although, MISTIE led to a mean reduction in hematoma size by 69% (SD 20) compared with 3% in the conservative treatment, no outcome benefit was found. At 12 months, 110 patients (45%) in the MISTIE group vs. 100 patients (41%) in the conservative group achieved a favorable outcome [adjusted risk difference 4% (95% CI − 4 to 12); *p* = 0.33]. The number of serious adverse events, such as symptomatic bleeding and cerebral infections, was similar between the two groups. The main conclusion of the study was that MISTIE is safe, but it does not improve long-term functional outcome. The authors performed a meta-analysis including only multisite trials of MISTIE in which functional outcome was evaluated by mRS or extended Glasgow Outcome Scale assessed at 180 days. No significant benefit of MISTIE was found (OR 0.61, 95% CI 0.29–1.26).

In summary, according to a large randomized, controlled, open-label, blinded endpoint phase 3 trial combined with a meta-analysis that compared minimally invasive surgery with thrombolysis vs. conservative management, despite being safe, it did not show long-term functional outcome benefit (Table 2) [47]. Therefore, MISTIE cannot be recommended as routine care in patients suffering from supratentorial ICH.

#### Ongoing trials on minimally invasive surgery

Minimally invasive surgery is an evolving area of interesting. Different techniques, new devices, and alternative approaches are being developed and tested. The Stereotactic Intracerebral Hemorrhage Underwater Blood Aspiration (SCUBA) technique has been tested in a cohort of 47 patients [52]. Because the SCUBA technique is performed in two phases, the first under dry-field conditions and the second using a wet-field strategy, it permits the surgeon to see the residual clot during hematoma drainage, and also it allows the visualization and cauterization of possible bleeding vessels. This technique has not been compared with other existing approaches.

There are several ongoing randomized clinical trials testing the benefits of other minimally invasive strategies, such as the ENRICH trial (Early Minimally-Invasive Removal of Intracerebral Hemorrhage), the INVEST (Minimally Invasive Endoscopic Surgical Treatment With Apollo/Artemis in Patients With Brain Hemorrhage), and the MIND (A Prospective, Multicenter Study of Artemis a Minimally Invasive Neuro Evacuation Device, in the Removal of Intracerebral Hemorrhage). These trials use different strategies for both patient inclusion criteria and evacuation methodology [53].

#### What does conservative treatment mean?

Since the early studies, conservative management means best medical care according to the best available evidence. Dr. Mckissoch and colleagues stated [31] that “we would stress that conservative treatment involves more than ‘doing nothing’; nursing care of a high standard, constant medical supervision, and control of cerebral edema and pulmonary complications are implicit in the term”. In the last five decades, our knowledge about this complex disease evolved. Currently, the best available evidence is summarized in documents, such as the American Heart Association/American Stroke Association *Guidelines for the Management of Spontaneous Intracerebral Hemorrhage* [9], the *European Stroke Organization (ESO) guidelines for the management of spontaneous intracerebral hemorrhage* [54], and the *Emergency Neurological Life Support: Intracerebral Hemorrhage* [23, 55].

Despite the fact that no drug or treatment have been shown to improve outcome after spontaneous ICH, there is robust evidence that patients suffering from ICH should be taken care in specialized neurological/neurosurgical intensive care units. Admission to a Neuro ICU is associated with decreased length of hospital stay and reduced mortality [56, 57], and might also be associated with improved functional outcome [58]. Additionally, transferring ICH patients to specialized Neuro ICU centers seems to be cost-effective [59].

It is also important to emphasize that premature care limitation or early withdrawal of life support, because of perceived poor prognosis may result in higher rates of mortality (i.e., self-fulfilling prophecy) [60]. Therefore, conservative management should mean initial aggressive medical management and ICU care, associated with (delayed) surgical evacuation if needed [18].

### Why surgical ICH trials may have failed?

- The primary injury of hemorrhage is not possible to be treated with surgery.
- Neurosurgical patients requiring urgent procedures are difficult to recruit.
- The ideal candidate and the optimal timing of surgery are essential questions that have not been determined [61].
- Many clinicians would consider hematoma drainage a life-saving measure in some situations; therefore, patients who were considered to benefit from surgery were not enrolled in these studies.
- Large crossover from medical management to surgical group. If no patient had crossed over from medical management to surgical group, the rates of unfavorable outcome and death with conservative management would have been higher.
- Problems with study designed, sample size, and number of excluded patients.
- Slow recruitment due to very restrictive inclusion protocols. A population-based study showed that very small percentages of ICH patients were eligible for the STICH II trial, i.e., 9.5% of lobar ICH without IVH and only 3.7% of all ICH patients [62].

### Special situations

#### Anticoagulant-associated intracranial hemorrhage

The use of anticoagulants increased the incidence of anticoagulant-related intracranial hemorrhage, which also increases the risk of hematoma expansion, unfavorable outcome, and death. In this clinical scenario, the reversal of drug effect is crucial, especially before surgical procedures [18, 63].

The management of intracranial hemorrhage associated with vitamin K antagonist includes the quick reversal of its effect by the use of prothrombin complex concentrates + vitamin K, with the ultimate goal of correcting the levels of international normalized ratio (keep INR < 1.3), within 4 h. Although the use of direct oral anticoagulants is associated with lower risk of ICH, the management of ICH associated with direct oral anticoagulant offers great challenge because it requires the use of specific antidotes not universally available. Platelet transfusions are not indicated in antiplatelet-associated ICH, unless a surgical procedure is foreseen [63, 64].

The Neurocritical Care Society and Society of Critical Care Medicine have published a *Guideline for Reversal of Antithrombotics in Intracranial Hemorrhage* [64]. Additionally, in these Thematic Series on *Acute Stroke Management* edited by Prof. Marek Mirski, Dr. Kuramatsu et al. have published a comprehensive review on this topic [63].

Pneumatic compression devices should be started on admission for venous thromboprophylaxis. Once the hematoma is radiologically stable in size for at least 24 h, pharmacological thromboprophylaxis with unfractionated heparin or low molecular weight heparin is recommended [65].

Some patients will require long-term oral anticoagulation resumption, especially those with mechanical heart or high-risk atrial fibrillation. The ideal timing of anticoagulation resumption is not well determined; however, ischemic complications are significantly higher when oral anticoagulation is not resumed in these patients [66].

#### Patients in coma (GCS score < 8), midline shift, large hematomas, or patients with refractory intracranial pressure

Decompressive craniectomy with or without hematoma evacuation may have a role for patients in coma with significant midline shift and large hematomas, or patients with refractory intracranial pressure. However, the available evidence of decompressive craniectomy is based on class III studies.

Fung et al. [67] evaluated the effect of decompressive craniectomy (150 mm + duraplasty) without hematoma evacuation in 12 consecutive patients with supratentorial ICH with median hematoma volume of 61.3 ml (interquartile range 37–83.5 mL), and median preoperative GCS score = 8 (interquartile range 4.3–10). The patients were matched with controls who were treated with conservative management. Three patients who underwent decompressive craniectomy died vs. 8 patients in the conservative group. Nine patients in the decompressive craniectomy group also had favorable outcome at 6 months according to the mRS (0–4). Decompressive craniectomy without hematoma drainage may also have a role in the setting of intracranial hemorrhage associated with refractory intracranial hypertension [68].

The use of decompressive craniectomy with hematoma drainage was also compared with hematoma drainage by craniotomy. Hayes et al. in a retrospective study compared hematoma evacuation ± decompressive craniectomy [69]. In the subgroup of patients with putaminal hemorrhage, ten patients underwent hematoma drainage with decompressive craniectomy and were compared with 16 patients who underwent hematoma drainage by craniotomy. Patients in the decompressive craniectomy group were more likely to have lower preoperative GCS (GCS < 8, *p* = 0.019). Decompressive craniectomy in putaminal hemorrhages was associated with a significant improvement in midline shift and a trend toward better outcome. In the subgroup of patients with lobar ICH, eight patients underwent hematoma drainage + decompressive craniectomy and 17 patients underwent only hematoma drainage by craniotomy. Patients in the decompressive craniectomy group were more likely to have larger midline shift (*p* = 0.022), and also were more likely to have right-sided hemorrhage (*p* = 0.011). No benefit of decompressive craniectomy was found in this subgroup of patients with lobar hemorrhages [69].

#### Surgical treatment of posterior fossa hemorrhage

Posterior fossa hemorrhage, i.e., bleeding taking place in the cerebellum or brainstem, is a severe life-threatening sub-type of ICH occurring in approximately 5 to 13% of all ICH cases [70]. Infratentorial compartment is very narrow and tight, which increases dramatically the risk of neurological deterioration due to progression in obstructive hydrocephalus (because of fourth ventricle compression) or local mass effect leading to compression on the brainstem. Infratentorial hemorrhages are an independent risk factor for mortality, regardless of hematoma volume [16]. There is no randomized controlled clinical trial comparing early surgical evacuation ± suboccipital decompressive craniectomy vs. conservative management for posterior fossa hemorrhage, and such a study is very unlikely to be performed [18]. Available management strategies, such as suboccipital decompressive craniectomy, EVD insertion for hydrocephalus management, or conservative management, are based on class III studies. These studies have suggested that cerebellar hemorrhages greater than 3 cm in diameter, or cerebellar hemorrhages compressing the brainstem compression or causing acute hydrocephalus may be better managed with early surgery [9]. The term early is also difficult to interpret in this patient population because the timing of surgery is not well established [18].

Patients with preserved level of consciousness (i.e., GCS 15 or 14) associated with cerebellar hematomas < 3 cm in diameter may be initially managed conservatively; however, in case of acute neurological deterioration (GCS ≤ 13), an urgent suboccipital craniectomy ± hematoma drainage should be performed [71, 72].

Other algorithms have been proposed. Da Pian et al. [73] were one of the first groups to study the effects of surgical management of posterior fossa hematomas. They performed a multicenter retrospective study in 22 Italian hospitals, including a total of 205 patients (155 cerebellar hematomas and 50 brainstem hematomas). Mortality was 38% for cerebellar hematomas vs. 57% for brainstem hematomas. In cerebellar hemorrhages, medical management was better when compared to surgical treatment, except for patients with hydrocephalus due to fourth ventricle obliteration or IVH. The level of consciousness 3 h after initial hemorrhage (i.e., awake patients) and the size of hematoma (< 3 cm) were significantly associated with better outcome. In brainstem hemorrhages, initial loss of consciousness and hematoma size were the main factors associated with outcome, regardless the presence of hydrocephalus. A total of 93% of patients with initial loss of consciousness, and 100% of comatose patients 3 h after the ictus experienced unfavorable outcome. According to the authors: “medical treatment appears to be the best policy for brainstem haematomas of limited size; for larger lesions (i.e., > 1.8cm), the outcome appears to be uniformly fatal, regardless of the treatment employed”.

Kirollos et al. developed a grading system based on the fourth ventricle size, configuration and location found in the CT scan [70]. Patients with a GCS ≥ 13 and a fourth ventricle Grade I (normal) and II (compressed or distorted) could be managed conservatively. In case of neurological deterioration, i.e., GCS < 13, in the presence of hydrocephalus, the authors suggest inserting an EVD, followed by hematoma evacuation if no clinical improvement. For patients with fourth ventricle Grade III (completely effaced), regardless of GCS, the authors suggest performing hematoma evacuation + CFS drainage [70].

More recently, Kuramatsu et al. [74] evaluated the impact of surgical hematoma evacuation on functional outcome after cerebellar hemorrhages. The authors performed an individual patient data meta-analysis of four observational ICH studies treated at 64 hospitals in the USA and Germany. The primary outcome was the proportion of patients with favorable outcome (mRS = 0–3) at 3 months. Secondary outcomes included the following: survival at 3 months, dichotomized functional outcome (mRS 0–3 vs 4–6) at 12 months, and survival at 12 months. From a total of 578 patients with cerebellar hemorrhage included in the database, 152 patients with surgical hematoma evacuation were matched by propensity score with 152 patients with conservative treatment. Hematoma evacuation was not associated with better functional outcome at 3 months (30.9% vs 35.5%, *p* = 0.39). However, hematoma evacuation was significantly associated with improved survival at 3 and 12 months (78.3% vs 61.2%, *p* = 0.001; 71.7% vs 57.2%, *p* = 0.008, respectively). The surgical evacuation of hematomas ≤ 12 ml was found to be harmful (reduced favorable functional outcome 30.6% vs 62.3%, *p* = 0.003), while the evacuation of hematomas ≥ 15 ml was robustly associated with improved survival (improved survival 74.5% vs 45.1%, *p* < 0.001) without a beneficial effect on functional outcome.

### Guidelines recommendations

According to the American Heart Association/American Stroke Association Guidelines for the Management of Spontaneous Intracerebral Hemorrhage [9] and the European Stroke Organization (ESO) guidelines for the management of spontaneous intracerebral hemorrhage [54], for the majority of patients with spontaneous supratentorial hemorrhage, the benefit of surgical evacuation is not well established (Class IIb; Level of Evidence A) [9], with no supporting evidence for routine surgery (moderate quality, weak recommendation) [54]. However, surgery may be lifesaving for patients with a GCS score 9–12 (moderate quality, weak recommendation) [54], or patients with delayed neurological deterioration (Class IIb; Level of Evidence C) [9].

Decompressive craniectomy with or without hematoma evacuation may reduce mortality in patients with putaminal ICH, especially in those in coma with large hematomas leading to significant midline shift, or also in patients with refractory intracranial hypertension (Class IIb; Level of Evidence C) [9].

Regarding the use of minimally invasive surgical approach, i.e., stereotactic or endoscopic aspiration with or without thrombolytic, its effectiveness remains uncertain (Class IIb; Level of Evidence B) [9].

Patients with posterior fossa hemorrhage with acute hydrocephalus, brainstem compression, or worsening in neuro status, surgery should be performed as soon as feasible (Class I; Level of Evidence B) [9].

## Conclusion

The role of open craniotomy for early hematoma drainage after intracranial hemorrhage remains a topic of hot debate. There is biological plausibility based on the prevention of cerebral herniation, the control of intracranial hypertension, and also avoidance or at least reduction in the impact of blood and its products on surrounding healthy tissue. However, randomized controlled trials failed to demonstrate this benefit in terms of mortality or functional outcome. Caution needs to be exercised when interpreting these results, because patients considered to benefit from surgery were excluded from the trials. Craniotomy for hematoma drainage remains a life-saving measure in critical situations. Additionally, minimally invasive techniques, such as neuroendoscopy or minimally invasive surgery with thrombolysis, despite being safe, are not associated with better long-term functional outcome. These minimally invasive techniques cannot be recommended as routine care in patients suffering from supratentorial ICH.

## Data Availability

Not applicable.

## Abbreviations

CSF: Cerebrospinal fluid
CT: Computed tomography
eGOS: Extended Glasgow outcome scale
EVD: External ventricular drain
CPP: Cerebral perfusion pressure
ICES: Intraoperative Computed Tomography–guided Endoscopic Surgery for Brain Hemorrhage
GCS: Glasgow Coma Scale
ICH: Intracranial hemorrhage
ICP: Intracranial pressure
IVH: Intraventricular hemorrhage
MISTIE: Minimally invasive catheter evacuation followed by thrombolysis
mRS: Modified Rankin scale
NIHSS: National Institutes of Health Stroke Scale
r-TPA: Recombinant tissue plasminogen activator
RR: Risk ratio
SBP: Systolic blood pressure
STICH: The International Surgical Trial in Intracerebral Hemorrhage
STICH II: Early surgery versus initial conservative treatment in patients with spontaneous supratentorial lobar intracerebral hematomas
